# A Multispectral Pulsed-Transmission Laser-Diode Sensor Concept for Real-Time In Situ Assessment of Microplastics in Water

**DOI:** 10.3390/s26144594

**Published:** 2026-07-20

**Authors:** Georgi V. Vladimirov, Ekaterina Iordanova, Georgi Yankov, Victoria Atanassova, Dimitar Filipov

**Affiliations:** 1 Georgi Nadjakov Institute of Solid-State Physics, Bulgarian Academy of Sciences, 1784 Sofia, Bulgaria; gjankov@issp.bas.bg (G.Y.); vatanassova@issp.bas.bg (V.A.); 2 AquaLID Ltd., 1784 Sofia, Bulgaria; dimitar.filipov@aqualid.eu

**Keywords:** microplastics, in situ optical sensing, multispectral transmission, laser-diode sensing, Urbach tail, extinction efficiency, direct-transmission probability, real-time water monitoring, active sensor volume, portable optical device

## Abstract

Microplastic monitoring needs methods that operate directly in water with minimal sample handling. Conventional techniques such as infrared and Raman spectroscopy and pyrolysis–GC/MS provide polymer-specific information but require sample preparation and delayed laboratory analysis. We propose an optical sensor concept for real-time, in situ microplastic assessment, based on multispectral pulsed transmission in the visible range using synchronized laser-diode lines and the directly transmitted signal through an active sensor volume. After calibration on particle-free water, each particle event reduces to a water-normalized transmission whose deficit is set by geometrical beam–particle overlap and the wavelength-dependent extinction efficiency. The weak polymer absorption is represented by the Urbach-tail formalism, the refractive-index-related redirection of light by a Fresnel-based, surface- and orientation-averaged probability of direct transmission, and particle size and shape are decoupled through an effective optical length. The coupled nonlinear system is solved for the bounds of the polymer absorption coefficient per candidate geometry. Because each polymer occupies a bounded region in multi-wavelength absorption space fixed by its band gap and structural state, the method can, in principle, separate structural modifications of identical composition, such as low- and high-density polyethylene. This is a sensor concept with a model-based proof of concept, not full environmental validation. Experimental verification on real reference particles is reported separately; the present article establishes the measurement model and inversion scheme that this verification builds on.

## 1. Introduction

Microplastic pollution has evolved into a major environmental and analytical challenge affecting freshwater systems, marine environments, soils, air, food chains, and human exposure pathways [1,2]. Microplastics are commonly defined as solid plastic particles with dimensions below 5 mm, although the practical lower detection limit depends strongly on the analytical method used [3]. Their relevance arises not only from their small size but also from their persistence, morphological variability, chemical heterogeneity, and ability to be transported, resuspended, ingested, and redistributed across environmental compartments. Because of these characteristics, microplastics are increasingly recognized as an issue of both ecosystem integrity and a human health concern [3,4,5,6]. Global plastics production reached 400.3 Mt in 2022, illustrating the scale of polymer material flows from which environmental microplastic contamination may arise [7,8].

A major challenge in microplastic monitoring is that the currently established analytical methods are predominantly laboratory-based. Fourier-transform infrared spectroscopy (FTIR), Raman spectroscopy, pyrolysis–gas chromatography–mass spectrometry (Py-GC/MS), scanning electron microscopy, and related methods have significantly advanced polymer-specific identification and particle characterization [9,10,11,12,13]. These methods differ in the type of information they provide. Spectroscopic techniques support particle-level polymer identification and morphology assessment, whereas thermal and chromatographic methods provide bulk chemical or mass-based polymer information. Even within spectroscopic approaches, manual, semi-automated, and automated FTIR/Raman workflows may differ in analysis time, operator dependence, and classification reliability [12]. However, these methods usually require sampling, filtration, extraction, digestion, drying, transport to a laboratory facility, instrumental analysis, and often extensive post-processing [9,11,12,14,15]. In addition, reliable microplastic analysis requires strict contamination control, blank correction, and quality assurance/quality control procedures, which further increases the complexity of routine monitoring [14,16]. Importantly, reported microplastic abundance can differ substantially depending on the analytical technique applied; for example, μFTIR and μRaman may produce markedly different particle counts, size distributions, and exposure estimates when applied to the same drinking-water sample set [15]. As a result, they are powerful reference methods but are less suitable for rapid field deployment, cost-efficient high-frequency monitoring, and continuous in situ observation [9,10,11,12,14,15,17,18].

These constraints become especially critical in dynamic aquatic environments, including rivers, reservoirs, industrial discharge streams, and drinking-water treatment systems, where both particle concentration and particle characteristics can change over time [19,20]. Riverine systems are especially challenging because the sampling strategy, hydrodynamic variability, particle transport, and sample representativeness strongly influence the measured microplastic abundance [19]. In such cases, a complementary method capable of operating directly in water, with minimal sample manipulation and rapid signal acquisition, would be of substantial practical value. Recent measurements of inherent optical properties of aqueous microplastic suspensions further indicate that optical-property datasets are important for developing and validating optical detection and quantification approaches [21]. The need is therefore not to replace validated laboratory methods, but to develop an optical approach that can provide continuous or near-real-time information in the field and support early screening, event detection, and trend monitoring [21,22,23,24]. This positions compact optical sensing as a complementary screening layer rather than as a replacement for confirmatory laboratory spectroscopy. Portable, continuous, and in situ optical-device concepts, including transmission/reflection, sizing-identification, and multiwavelength configurations, further support the feasibility of compact field-oriented detection [22,25,26,27]. More broadly, in situ optical water-quality monitoring based on absorbance, scattering, reflectance, and fluorescence measurements is increasingly used for continuous monitoring, although matrix effects and calibration remain key challenges [28].

Advances in multiwavelength laser-source technology provide a promising basis for compact optical assessment of suspended microplastics. In particular, previous work based on metal-vapor laser emission demonstrated that the use of several visible wavelengths can improve the spectral dimensionality of the optical response and increase the possibility of distinguishing different polymer classes [29]. In that study, laser operation was demonstrated not only at the three visible lines 510.6, 578.2, and 627.8 nm but also with an additional 312.2 nm Au line and nine further lines obtained by adding Fe and Pb active particles and by applying nonlinear frequency conversion, indicating the potential use of up to thirteen discrete laser lines for green-technology applications [29]. The use of a larger number of discrete spectral lines can provide substantially richer information about particle-dependent optical behavior, including wavelength-dependent attenuation trends, polymer-specific response contrast, and geometry-related variations. However, increasing the number of spectral channels also increases the complexity of the optical source, beam-combining architecture, detection scheme, synchronization, alignment, and signal processing. That study demonstrated the feasibility of using multiple discrete laser lines for polymer-dependent optical response assessment, but the present work shifts the device concept toward synchronized laser-diode implementation for compact in situ use. At the same time, practical device design requires a balance between spectral information and instrumental simplicity. For this reason, the present work is focused not on maximizing the number of spectral channels, but on identifying a compact and realistic architecture based on only three synchronized visible laser-diode lines as a practical compromise for a small, portable, field-deployable device.

The objective of the present article is to establish the theoretical and methodological basis of a compact optical method for real-time, in situ assessment of suspended microplastics in water. The proposed concept is based on multispectral pulsed-transmission measurements in the visible spectral range, where water remains sufficiently transparent and the weak optical absorption of polymer particles can be interpreted using the Urbach-tail formalism. The central idea is that, after calibration on particle-free water, the water-normalized transmission of a single particle is governed by two separable contributions—a geometrical beam–particle overlap and a wavelength-dependent extinction efficiency—and that a small set of synchronized wavelengths is sufficient to invert this relation. The scientific focus of this article is therefore placed on the measurement model, the extinction-efficiency formulation, the Fresnel-based, surface- and orientation-averaged effective probability of direct transmission, the decoupling of size and shape through an effective optical length, and the coupled nonlinear inversion that recovers the bounds of the polymer absorption coefficient.

The novelty of this work lies in the integration of a transmission-only optical geometry, a synchronized three-line pulsed diode-laser architecture, and a defined active sensor volume with a physically grounded inversion scheme that separates material from geometry. Unlike laboratory spectroscopic workflows that rely on sample extraction and broadband spectral acquisition, the proposed concept evaluates directly transmitted pulse responses during particle transit through the irradiated volume, recovers the wavelength-dependent absorption coefficient by solving a coupled nonlinear system, and assigns each particle to the per-polymer region it occupies in multi-wavelength absorption space. Because that region is fixed by the band-gap and structural state of the polymer rather than by its molecular vibrational fingerprint, the method offers, in principle, the ability to separate structurally distinct but chemically identical polymers such as low- and high-density polyethylene—a discrimination that is difficult for standard vibration-based polymer-identification workflows.

A further consequence of this architecture concerns particle loading. Reported microplastic number concentrations in freshwaters and drinking water are highly variable but, across water types, have been compiled in critical reviews to span roughly ten orders of magnitude, from about $10^{-2}$ to $10^{8}$ particles per cubic meter [30]. For the compact active sensor volume used here—a beam of 3–5 mm diameter over an optical path of the order of one meter, i.e., only about $10^{-5}$ m^3^ (roughly 7–20 mL)—even an abundance as high as $10^{4}$ particles per cubic meter (about ten particles per litre, near the upper range reported for drinking and surface waters) corresponds to far fewer than one particle inside the volume at any instant. Each interaction event therefore involves, to a very good approximation, a single particle, which is the regime assumed throughout the model; only heavily loaded streams approaching the extreme upper end of reported concentrations would require dilution or a smaller active volume to preserve this condition.

## 2. Materials and Methods

### 2.1. Measurement Regime, Active Sensor Volume, and Working Assumptions

The proposed method operates in a geometrical-optics, direct-transmission, pulsed multispectral measurement regime implemented through a single measuring channel. The optical interrogation is performed in the visible spectral range, where the attenuation of pure water is sufficiently low over the short path lengths required for compact flow-through sensing [31]. The wavelength-dependent polymer absorption response is represented using the Urbach-tail formalism [32,33]. This regime is justified by the large size parameter of the particles of interest: for a particle dimension d from tens of micrometers upward at visible wavelengths, the size parameter x=πd/λ is of order $10^{3}$–$10^{4}\gg 1$, so the ray-optics (anomalous-diffraction) description in terms of an effective projected area, an effective optical length, a Fresnel reflectance, and an extinction efficiency is valid, and a full electromagnetic scattering solution is not required for each event [34,35,36]. The theoretical target range extends from tens of micrometers to the sub-millimeter scale. Its lower bound is set by the beam diameter, alignment, and signal-to-noise ratio; in the present proof-of-concept diode-laser geometry with a beam diameter of approximately 3.5 mm, the practically measurable lower-size range is expected to be closer to 250–300 µm, corresponding to an overlap ratio σp/σb of the order of 0.01; smaller particles would require a reduced beam diameter, improved alignment, or a multi-beam configuration, with a beam of about 1 mm extending the lower limit toward roughly 100 µm. Its upper bound is set by the requirement that the particle be smaller than the beam, as stated in the working assumptions below. The relevant invertibility criterion is that the particle be inscribable within the beam cross-section, so that 0 ≤ σp/σb < 1; with the present beam, any particle that fits within a sphere of the beam diameter is invertible, and elongated fragments or fibers with one small transverse dimension therefore remain measurable across much of the millimeter range. The beam diameter is a tunable engineering parameter rather than a fundamental limit: additional beam-shaping optics extend the beam toward the upper size of interest, widening the measurable window at the cost of power density and signal-to-noise ratio.

**Figure 1:**
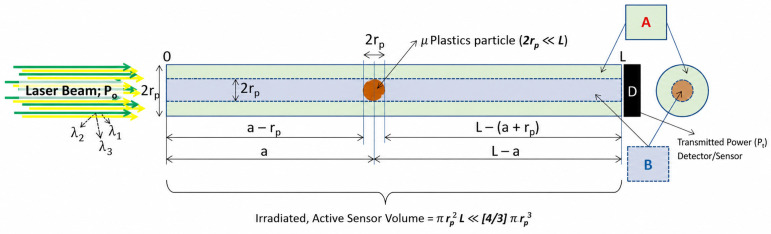
Measurement geometry (single measuring channel). The three-wavelength laser beam (incident power P_0_; lines λ_1_, λ_2_, λ_3_; radius r_b_) crosses a water channel of optical path length L; a suspended particle (radius r_p_) at axial position a (distinct from the Urbach spectral-slope parameter a of Section 2.4) intersects the beam, and the directly transmitted power P_t_ is collected by detector D. The irradiated active sensor volume is π·rb^2^·L.

The active sensor volume is defined as the spatial region where the combined multispectral laser beam intersects the water channel and from which the directly transmitted optical component is accepted by the detection path. A suspended microplastic particle is considered measurable when it crosses this irradiated volume and modifies the optical power that remains aligned with the original beam-propagation axis. The detector is therefore not intended to collect the full angular distribution of scattered, reflected, or diffracted light. Optical power redirected outside the detector acceptance path is treated as a loss from the direct-transmission channel and is included in the effective particle-related extinction response. Figure 1 shows this single-channel transmission geometry, where only the power that stays aligned with the beam axis reaches detector D and the redirected light is counted as particle extinction.

The measurement model is based on the following working assumptions. First, the actual particle-free background of the water under test is measured as a calibrated optical baseline and is assumed to remain stable over the calibration-to-event interval; the low intrinsic attenuation of pure water sets the favorable order of magnitude [31], but the operational baseline is that of the measured matrix rather than of ideal water. Second, the refractive index of water $n_{w}$ is taken as known and only weakly dispersive across the three selected lines, consistent with its use in the direct-transmission probability of Section 2.5. Third, the particle is assumed to be smaller than the beam cross-section ($r_{b}$ larger than the particle dimensions), so that $0\leq \sigma_{p}/\sigma_{b}<1$ and the geometry closure of Section 2.6 applies; particles comparable to or larger than the beam fully obstruct it ($\sigma_{p}/\sigma_{b}\to 1$) and fall outside the present invertible regime, which with the 3.5 mm proof-of-concept beam, restricts the upper measurable size to below roughly one millimeter. Fourth, the suspension is sufficiently dilute that one dominant particle interacts with the active sensor volume during a single event; overlapping multi-particle events and strong aggregation are outside the primary operating model. Fifth, the particle orientation remains effectively unchanged during an individual nanosecond pulse, although its projected cross-section and optical length may vary during the complete crossing event. Sixth, the measured response is not interpreted as a single-value chemical identifier; instead, each particle event is represented by a sequence of normalized wavelength-dependent transmission measurements acquired while the particle passes through the active sensor volume.

The overall strategy of the method is to separate the contribution of the polymer substance, expressed through the wavelength-dependent absorption coefficient, from the contribution of particle geometry, expressed through the projected cross-section and the effective optical length. This separation is what ultimately allows the method to assign a particle to a polymer class, and it defines the structure of all subsequent subsections: the measured quantity (Section 2.2), the extinction model (Section 2.3), the absorption formalism and its physical justification (Section 2.4), the direct-transmission probability (Section 2.5), the geometry formalism (Section 2.6), and the numerical inversion that ties them together (Section 2.7). In the proof-of-concept laboratory configuration, the reference particles are presented in a controlled orientation for calibration (Section 2.9), whereas the in-flow crossing regime described above applies to deployment; the two share the same response model and differ only in how the sequence of projected cross-sections and optical lengths is sampled.

### 2.2. Measured Quantity: Water-Calibrated Normalized Transmission

The measurement is first defined at the level of directly recorded pulse signals. For each selected wavelength $\lambda_{i}$ and for each discrete pulse index k during a particle-crossing event, the system records an incident (reference) optical pulse $P_{0,i,k}\left(t\right)$ and a transmitted optical pulse $P_{tr,i,k}\left(t\right)$. Because the system operates with nanosecond pulsed emission, the primary measured quantity for each pulse is the pulse energy obtained by integrating the corresponding pulse-power signal over the pulse duration $t_{p}$:

(1) $$E_{0,i,k}=\int_{0}^{t_{p}}P_{0,i,k}\left(t\right)\;dt$$

(2) $$E_{tr,i,k}=\int_{0}^{t_{p}}P_{tr,i,k}\left(t\right)\;dt$$

This formulation allows pulse-to-pulse comparison even when small fluctuations occur in the emitted pulse amplitude.

The measurement proceeds in two stages that are essential to understanding what the system actually measures. In the first stage, a reference measurement is performed with particle-free water. This water baseline accounts for the wavelength-dependent attenuation in the water path, the fixed optical losses in the channel, and the stable transmission response of the optical system. The water-only transmission term is

(3) $$T_{w}\left(\lambda_{i}\right)=e^{-K_{a}\left(\lambda_{i}\right)\;L}$$

where $K_{a}\left(\lambda_{i}\right)$ is the attenuation coefficient of water at wavelength $\lambda_{i}$, characterized by the established pure-water absorption spectrum [31], and L is the optical path length through the water channel. For the present configuration and water quality, the wavelength dependence of the water refractive index is taken as weak and monotonic in the visible range, so averaged literature values are sufficient at this stage.

In the second stage, particles are introduced, and the transmitted pulse energies are measured. The particle-related, water-calibrated normalized transmission for each pulse is then obtained by correcting the transmitted pulse energy for the incident pulse energy and for the water-only attenuation:

(4) $$T_{m}\left(\lambda_{i},k\right)=\frac{E_{tr,i,k}}{E_{0,i,k}\;e^{-K_{a}\left(\lambda_{i}\right)\;L}}$$

In this form, $T_{m}\left(\lambda_{i},k\right)$ is the experimentally accessible quantity that contains the particle contribution only: the deviation of $T_{m}$ from unity is what the particle adds to (removes from) the directly transmitted beam after the water background and the incident-pulse variation have been removed. This is precisely the design intent of the two-stage scheme—calibrate on water, then measure with particles, so that the evaluated signal is attributable to the particle and not to the laser output, the alignment, the detector response, or the water path.

The observable used for further evaluation is therefore the multispectral sequence

(5) $$T_{m}\left(k\right)=\{T_{m}\left(\lambda_{1},k\right),\;T_{m}\left(\lambda_{2},k\right),\;T_{m}\left(\lambda_{3},k\right)\}$$

acquired repeatedly while the particle crosses the active sensor volume. Additional descriptors—pulse amplitude, pulse width, integrated energy, and relative timing shift—may be extracted from the same recorded signals, but the normalized transmission sequence is the primary quantity that connects the measurement to the optical-response model.

### 2.3. Particle Extinction Model

After normalization to the water baseline, the remaining signal variation is attributed to the interaction between the particle and the directly transmitted beam. The water-calibrated transmission at each wavelength is related to the particle through the geometrical beam–particle overlap and the extinction efficiency of the particle:

(6) $$T_{m}\left(\lambda_{i}\right)=1-\frac{\sigma_{p}}{\sigma_{b}}\;Q_{ext}\;\left(n,\alpha \left(\lambda_{i}\right),d_{op}\right)$$

where $\sigma_{p}$ is the projected geometrical cross-section of the particle, $\sigma_{b}$ is the geometrical cross-section of the laser beam, and $Q_{ext}$ is the dimensionless extinction efficiency of the particle. The ratio $\sigma_{p}/\sigma_{b}$ is the fraction of the beam cross-section that is geometrically affected by the particle; for an irregular particle, this ratio varies from pulse to pulse as the projected area changes during the crossing. Because the detector accepts only the on-axis directly transmitted component, any light redirected off-axis—including by diffraction and edge effects—falls outside the acceptance path and is registered as a loss from the direct channel; it is therefore included in the measured extinction and in the probability of direct transmission (Section 2.5) rather than neglected.

The extinction efficiency $Q_{ext}$ is not absorption alone. In the present transmission-only configuration, it represents the total fraction of light removed from the directly detected beam component within the affected part of the beam, and it depends on three quantities: the real part of the refractive index n, the wavelength-dependent absorption coefficient $\alpha \left(\lambda \right)$, and the effective optical length of the particle along the propagation direction $d_{op}$,

(7) $$Q_{ext}=f_{Q}\;\left(n,\alpha \left(\lambda \right),d_{op}\right)=1-\frac{{\left(1-R\right)}^{2}e^{-\alpha \left(\lambda \right)\;d_{op}}}{1-R^{2}\;e^{-2\alpha \left(\lambda \right)\;d_{op}}}$$

where R is the reflectance at the particle surface, set by the refractive-index contrast through the Fresnel relations of Section 2.5; the factor ${\left(1-R\right)}^{2}e^{-\alpha d_{op}}$ is the fraction transmitted directly after entry and exit with single-pass absorption, and the denominator $1-R^{2}e^{-2\alpha d_{op}}$ accounts for multiple internal reflections. In the weak-reflection, sub-gap-absorption regime of interest, this separates into a refractive-index-related redirection of light out of the direct beam, carried by the probability of direct transmission (Section 2.5), and an absorption-related attenuation along the optical path, carried by the Urbach-tail absorption (Section 2.4). Equation (7) in this closed form holds strictly only for an idealized particle whose entry and exit faces are parallel to each other and perpendicular to the propagation direction, so that the directly transmitted ray stays collinear with the incident beam; real microplastic surfaces depart from this ideal, and the realistic, orientation-averaged treatment of the resulting redirection of light is exactly the role of the probability of direct transmission introduced in Section 2.5. The closed-form Equation (7) therefore serves only to motivate the separation of index-related redirection from absorption; it is not used directly in the inversion, and the plane-parallel-plate assumption on which it rests does not enter the recovered absorption bounds, which are carried entirely by the orientation-averaged PDTeff of Section 2.5. This formulation follows the established description of extinction by particles in terms of an efficiency factor rather than an explicit angular scattering calculation for each event [34,35]. It separates cleanly into a refractive-index-related redirection of light out of the direct beam, treated through the probability of direct transmission introduced in Section 2.5, and an absorption-related attenuation along the optical path, treated through the Urbach-tail absorption introduced in Section 2.4.

The model is used within physically bounded parameter ranges,

(8) $$0\leq \frac{\sigma_{p}}{\sigma_{b}}\leq 1,\;\;0\leq Q_{ext}\leq 1,\;\;0\leq T_{m}\leq 1,\;\;\alpha \left(\lambda \right)\geq 0$$

which ensure that the normalized transmitted signal remains physically meaningful and non-negative. In particular, $\sigma_{p}/\sigma_{b}$ must be interpreted as an effective overlap factor: if the projected particle area exceeds the beam area, the ratio is limited by the part of the beam actually affected by the particle.

Equation (6) is the central observable of the method. It shows that a single transmission value cannot uniquely determine the polymer type, size, shape, and orientation, because it couples a geometrical contribution ($\sigma_{p}/\sigma_{b}$) with a material-and-geometry contribution ($Q_{ext}$). The role of the subsequent sections is to provide enough physical structure—and enough wavelength samples—to invert this coupling.

### 2.4. Wavelength-Dependent Polymer Absorption and Its Physical Justification

**Figure 2:**
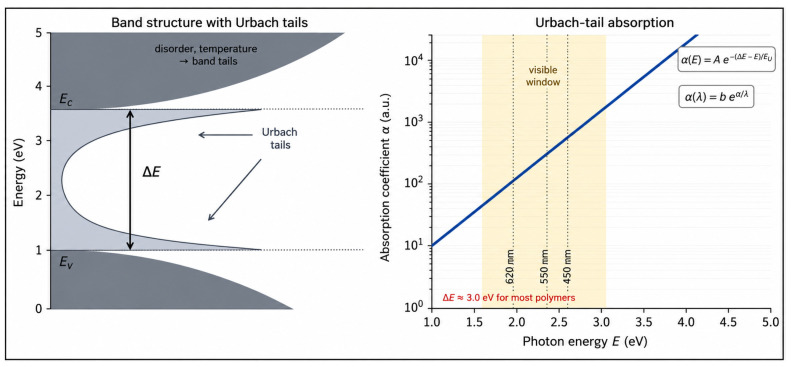
Urbach-tail picture. Left: band structure in which disorder and temperature broaden the band edges into exponential Urbach tails that reduce the effective gap below ΔE. Right: the resulting sub-gap absorption is linear on a logarithmic scale (the Urbach rule), $\alpha \left(E\right)=A\;e^{-\left(\Delta E-E\right)/E_{U}}$, and equivalently $\alpha \left(\lambda \right)=b\;e^{a/\lambda }$; the shaded band marks the visible window (about 1.55–3.10 eV, 800–400 nm) and the dotted lines the three measurement wavelengths. For most polymers, ΔE≫3.0 eV, so the visible range probes the smooth tail rather than the band edge.

The extinction model requires a wavelength-dependent absorption coefficient $\alpha \left(\lambda \right)$ for the polymer particle. In the visible range (approximately 400–800 nm), the optical band gap of common polymers is large (ΔE≫3 eV), so the spectral region of interest lies well within the sub-gap absorption tail rather than across narrow vibrational bands. In this region, the dominant mechanism is Urbach-tail absorption [32,33], in which the absorption coefficient depends exponentially on photon energy,

(9) $$\alpha \left(E\right)=A\;exp\;\left(-\frac{\Delta E-E}{E_{U}}\right)$$

where A is a pre-exponential coefficient, ΔE is the effective absorption-edge (band-gap-related) energy, $E_{U}$ is the Urbach energy describing the width of the tail, and E is the photon energy. Using E=hc/λ, the same relation is written in the optically convenient wavelength form

(10) $$\alpha \left(\lambda \right)=b\;exp\;\left(\frac{a}{\lambda }\right)$$

where a and b are fitting parameters describing the smooth wavelength-dependent absorption trend of the polymer in the selected visible range: a controls the spectral slope, and b scales the magnitude of the absorption coefficient. Recovering a and b from the multispectral measurement is therefore equivalent to recovering $\alpha \left(\lambda \right)$. The physical origin of this behavior is illustrated in Figure 2.

The validity of inverting a transmission measurement for $\alpha \left(\lambda \right)$ while treating n as known is supported by the Kramers–Kronig relations, which link the real and imaginary parts of the complex refractive index $\tilde{n}\left(\omega \right)=n\left(\omega \right)+i\kappa \left(\omega \right)$:

(11) $$n\left(\omega \right)-1=\frac{2}{\pi }\;P\;\;\int_{0}^{\infty }\frac{\Omega \;\kappa \left(\Omega \right)}{\Omega^{2}-\omega^{2}}\;d\Omega$$

(12) $$\kappa \left(\omega \right)=-\frac{2\omega }{\pi }\;P\;\;\int_{0}^{\infty }\frac{n\left(\Omega \right)-1}{\Omega^{2}-\omega^{2}}\;d\Omega$$

with $\alpha \left(\lambda \right)=4\pi \kappa \left(\lambda \right)/\lambda$. Because n and κ are not independent, a smooth, monotonic visible-range absorption tail is consistent with a slowly varying real index, which justifies treating n as weakly dispersive and known to first order while solving for the absorption parameters, consistent with reported visible-range refractive-index data for common polymers [37,38].

A central consequence of this formalism is that the chemical composition of a polymer fixes its absorption only within bounded limits. The band gap ΔE and the Urbach energy $E_{U}$ are set by the molecular composition and the structural state of the material. Individual particles of the same polymer differ in crystallinity, additives, defects, ageing, surface condition, and local micro-disorder, and therefore exhibit a spread of $\left(a,b\right)$ values; this spread, however, remains confined to a region determined by composition and structure [37,38,39,40,41]. Plotting the recovered α against the three sampled wavelengths therefore maps each measurement to a point in a three-dimensional absorption space, and each polymer occupies a bounded region (a “volume”) in that space. The exact position of a given particle within its region depends on its geometry and structural state, while the region itself is characteristic of the polymer.

This picture has an important practical implication. Two materials with an identical chemical composition but different structure—such as low-density and high-density polyethylene—share essentially the same primary molecular vibrational fingerprint, so that standard FTIR and Raman workflows typically classify both as polyethylene and resolve the structural difference only with additional crystallinity-sensitive band analysis or chemometric processing. Their band-gap and disorder differ, however, so they occupy distinct regions in the multi-wavelength absorption space sampled here. The proposed method can therefore, in principle, separate such structural modifications, which is a capability that complements rather than reproduces the chemical specificity of FTIR, Raman, or Py-GC/MS.

The use of three wavelengths follows directly from this structure. With one wavelength, transmission cannot separate material absorption from the projected area and optical length. With two wavelengths, a relative spectral trend can be estimated but geometry and attenuation remain coupled. Three wavelength samples provide the minimal basis needed to constrain the two absorption parameters $\left(a,b\right)$ together with the geometrical unknowns while preserving a compact diode-laser architecture. In the proposed implementation, the selected wavelengths are 490 nm, 520 nm, and 640 nm; they are not intended to reconstruct a full absorption spectrum but to provide a compact, physically interpretable signature in the reduced absorption space. The specific choice of three lines follows from two independent constraints. First, source availability: in the visible window where water is transparent, practical nanosecond-pulsed sources are limited to metal-vapor lasers (the 510.6/578.2/627.8 nm lines of the copper-vapor source used in earlier work of this group [29]) and combinations of discrete laser diodes (490/520/640 nm here); the wavelengths are therefore selected from what compact, synchronizable pulsed sources actually make available rather than being freely optimized. Second, the sub-gap absorption follows the two-parameter Urbach form α(λ) = b·exp(a/λ), so three lines are the minimal set that over-determines the two absorption parameters together with the geometrical unknowns while keeping the source compact; additional visible lines improve accuracy but the alignment, synchronization, and calibration burden beyond three to four synchronized beams outweighs the gain. Within this triplet, the LDPE/HDPE contrast is carried predominantly by the 640 nm channel, consistent with the polymer-dependent response reported for the neighboring metal-vapor lines in [29].

### 2.5. Probability of Direct Transmission

The refractive-index-related part of $Q_{ext}$ is described by the probability of direct transmission, $PDT_{eff}$, which is the probability that light entering the particle leaves it still aligned with the direct beam, i.e., that it is transmitted through the entry and exit interfaces without being reflected out of the detected component. This quantity is needed precisely because the idealized parallel-face geometry assumed by the closed form of Equation (7) is essentially never met in practice: the entry and exit microsurfaces of a real particle are tilted and curved, so that even small deviations from parallel faces perpendicular to the beam redirect part of the light out of the measured forward direction. This redirection is readily observed: an illuminated particle does not simply cast a clean shadow but visibly scatters light in all directions, so that it appears to glow. $PDT_{eff}$ is the angle-averaged probability that a ray nevertheless leaves the particle still aligned with the direct beam, which is why it lies below unity and is supplied to the inversion as a bounded, geometry-related preliminary quantity rather than as the idealized slab transmission of Equation (7). For a particle in water with refractive index $n_{p}$ surrounded by water of index $n_{w}$, this probability is constructed from the Fresnel reflectances at the two interfaces and averaged over the distribution of local incidence angles that represents the particle surface:

(13) $$PDT_{eff}\left(\lambda \right)=\frac{1}{N}\sum_{i=1}^{N}\left(1-R_{en,i}\right)\left(1-R_{ex,i}\right)$$

where $R_{en}$ and $R_{ex}$ are the reflectances at the entry and exit interfaces. Although the diode-laser emission is linearly polarized, the average in Equation (13) is taken over a broad distribution of local incidence planes and angles set by the particle surface, which randomizes the orientation of the polarization relative to each local interface; the polarization-averaged (unpolarized) Fresnel form is therefore used as an effective reflectance, with each reflectance given by

(14) $$R=\frac{1}{2}\left[{\left(\frac{sin\left(\theta_{1}-\theta_{2}\right)}{sin\left(\theta_{1}+\theta_{2}\right)}\right)}^{2}+{\left(\frac{tan\left(\theta_{1}-\theta_{2}\right)}{tan\left(\theta_{1}+\theta_{2}\right)}\right)}^{2}\right]$$

and the refraction angles follow from Snell’s law at the water–particle and particle–water interfaces,

(15) $$\theta_{en}=arcsin\;\left(\frac{n_{w}}{n_{p}}sin\theta_{n}\right),\;\;\theta_{ex}=arcsin\;\left(\frac{n_{p}}{n_{w}}sin\theta_{x}\right)$$

Equations (13)–(15) make explicit how $PDT_{eff}$ depends on substance and on geometry, which is the point that must be stated clearly rather than assumed. The dependence on substance enters only through the refractive-index contrast $n_{p}/n_{w}$; the dependence on geometry and surface state enters through the distribution of incidence angles $\theta_{n}$ over which the average in Equation (13) is taken—a smooth, near-spherical surface samples mostly near-normal angles and yields a high transmission probability, while a rough or irregular surface samples a broad angular range and lowers it. Because the refractive index of common polymers varies only weakly across the selected visible lines, $PDT_{eff}$ has a weak dispersion and changes little between polymers; in a first approximation, it can be evaluated with averaged refractive-index values, and the angular average is estimated by Monte-Carlo sampling of randomly generated incidence angles consistent with the assumed surface roughness. $PDT_{eff}$, therefore, enters the inversion (Section 2.7) as a preliminary, bounded quantity rather than as a free fitting parameter, and it replaces the explicit reflection factor of a smooth-surface model with an effective coefficient appropriate to the direct-transmission geometry. It should be emphasized that PDTeff is not a competing unknown of the same kind as the absorption parameters: because the device measures only the on-axis directly transmitted power, PDTeff is precisely the bounded, pre-computable measure of the light redirected out of that channel by reflection, refraction, and scattering at the particle microsurfaces. Its value is dominated by the refractive-index contrast np/nw and by the ordering of the entry and exit microsurfaces over a single pass, and the Monte-Carlo average converges to a stable per-polymer value once the number of sampled microsurfaces exceeds about 10^4^ (for a millimeter-scale particle at a microsurface resolution of the order of tens of micrometers). Because the resulting PDTeff band (approximately 0.85–0.90 for the polyolefins) is narrow relative to the much larger inter-polymer spread in absorption, supplying PDTeff as a bounded preliminary quantity shifts the recovered bounds only slightly rather than re-introducing the geometric unknowns into the class assignment; for strongly irregular or aged surfaces, this band widens and must be re-characterized during calibration.

**Figure 3:**
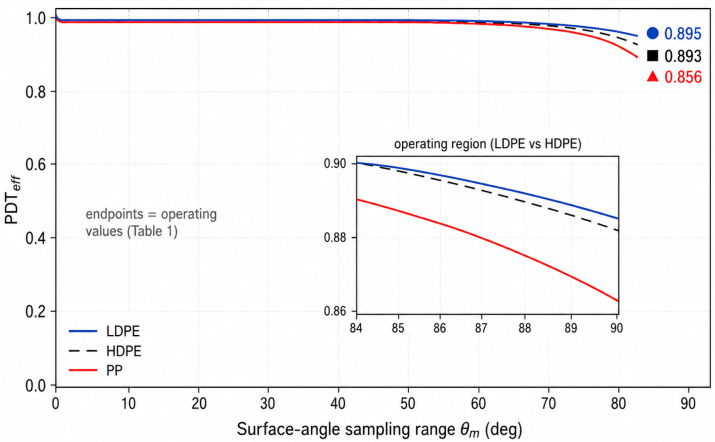
Direct-transmission probability for LDPE, HDPE, and PP versus the surface-roughness angular range. $PDT_{eff}$ falls from unity for a smooth surface to the operating value adopted for each polymer (Table 1) over the full orientation range; PP lies distinctly below the two polyethylenes, which nearly coincide and are separated instead by their absorption (Figures 7 and 8).

The dependence of $PDT_{eff}$ on the surface-roughness range is shown in Figure 3 for the three polyolefins, where each curve falls toward the operating value of Table 1 and PP lies distinctly below the two polyethylenes.

### 2.6. Geometry Formalism: Decoupling Size and Form

The geometrical part of the model explains how particle size, orientation, and trajectory influence the normalized transmission sequence, and it is what allows size and shape to be decoupled from substance. A particle crossing the active sensor volume is treated not as a static object producing one fixed attenuation value, but as a moving optical object whose projected cross-section and effective optical length may change from pulse to pulse during the same crossing event.

**Figure 4:**
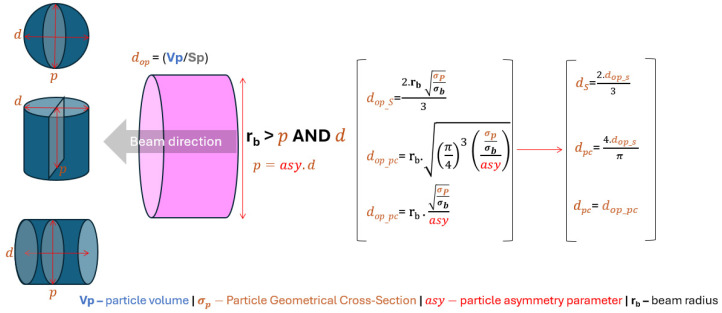
Geometry formalism (“size to form”): a particle of transverse and longitudinal dimensions d and p=asy⋅d presents different projected cross-sections $\sigma_{p}$ and optical lengths $d_{op}=\left(V_{p}/\sigma_{p}\right)$ depending on its shape (sphere, cylinder, disk) and orientation relative to the beam, with shape-specific relations linking $d_{op}$ to $\sigma_{p}/\sigma_{b}$ (valid for $r_{b}>p$ and d).

The effective optical length is related to the particle volume and the projected cross-section perpendicular to the beam through the shape relation used in the model,

(16) $$d_{op}=\frac{V_{p}}{\sigma_{p}}\;$$

where $V_{p}$ is the particle volume and $\sigma_{p}$ the projected cross-section. Equation (16) expresses, within the shape parameterization of the model, how the effective optical path follows the volume-to-area ratio of the particle; the concrete normalization for each assumed shape class is fixed by the relations below (Equation (17) for a sphere, with analogous expressions for disk- and cylinder-like shapes). This definition expresses the key physical fact that the same particle presents different optical lengths—and therefore different absorption at each wavelength—when its orientation relative to the beam changes. Particle shape is parameterized by an asymmetry parameter through p=asy⋅d, where p and d are the longitudinal and transverse dimensions, and the optical length is related to the measurable overlap ratio for each shape class. For a sphere, for example,

(17) $$d_{op,S}=\frac{2\;r_{b}}{3}\sqrt{\frac{\sigma_{p}}{\sigma_{b}}}$$

with $r_{b}$ the beam radius, and analogous relations hold for disk- and cylinder-like shapes in different orientations (provided the beam is larger than the particle, $r_{b}>p$ and $r_{b}>d$). For each assumed shape class, the geometry therefore supplies a closure relation between the overlap ratio and the effective optical length,

(18) $$\frac{\sigma_{p}}{\sigma_{b}}=f_{geom}\left(\beta \right)$$

where β is the effective optical length set by the particle’s shape and orientation—the quantity d_op of Equations (16) and (17) as it enters the inversion; the dimensionless shape asymmetry is denoted *asy*. Equation (18) is the closure that reduces the number of independent geometrical unknowns and makes the inversion of Section 2.7 well posed for a given candidate geometry. The way particle shape and orientation set $\sigma_{p}$ and $d_{op}$ is illustrated in Figure 4.

### 2.7. Numerical Inversion: Recovering the Absorption Bounds

Section 2.2, Section 2.3, Section 2.4, Section 2.5 and Section 2.6 together define a coupled system in which the measured quantities $T_{m}\left(\lambda_{i}\right)$ are connected to the polymer absorption parameters $\left(a,b\right)$, the geometrical parameters $\left(\beta ,\sigma_{p}\right)$, and the preliminary direct-transmission probability $PDT_{eff}\left(\lambda_{i}\right)$. To make the dependence explicit, an intermediate per-line quantity is introduced,

(19) $$A\left(\lambda_{i}\right)=\frac{PDT_{eff}\left(\lambda_{i}\right)}{1-\frac{1-T_{m}\left(\lambda_{i}\right)}{\varphi }}$$

where φ is the effective beam–particle overlap (geometrical filling) factor that relates the global transmission deficit $1-T_{m}$ to the local extinction within the part of the beam covered by the particle; within the bounded model, φ is identified with $\sigma_{p}/\sigma_{b}$ and is solved jointly. With the Urbach form $\alpha \left(\lambda \right)=b\;e^{a/\lambda }$, the three measured lines $\lambda_{1},\lambda_{2},\lambda_{3}$ give the coupled nonlinear system

(20) $$exp\;\left(a\;\frac{\lambda_{2}-\lambda_{1}}{\lambda_{2}\lambda_{1}}\right)=\frac{lnA\left(\lambda_{1}\right)}{lnA\left(\lambda_{2}\right)}$$

(21) $$exp\;\left(a\;\frac{\lambda_{3}-\lambda_{2}}{\lambda_{3}\lambda_{2}}\right)=\frac{lnA\left(\lambda_{2}\right)}{lnA\left(\lambda_{3}\right)}$$

(22) $$\beta \;b\left(e^{a/\lambda_{1}}-e^{a/\lambda_{2}}\right)=ln\frac{A\left(\lambda_{1}\right)}{A\left(\lambda_{2}\right)}$$

(23) $$\beta \;b\left(e^{a/\lambda_{2}}-e^{a/\lambda_{3}}\right)=ln\frac{A\left(\lambda_{2}\right)}{A\left(\lambda_{3}\right)}$$

closed by the geometry relation $\sigma_{p}/\sigma_{b}=f_{geom}\left(\beta \right)$ of Equation (18). The measured transmissions $T_{m}\left(\lambda_{i}\right)$ are known, $PDT_{eff}\left(\lambda_{i}\right)$ is evaluated as a bounded preliminary quantity (Section 2.5), and the unknowns are the absorption parameters a and b together with the geometrical parameters β and $\sigma_{p}$. Here, β is the effective optical length (the quantity d_op_ of Equations (16) and (17)) as it enters the inversion, while the dimensionless shape asymmetry is denoted *asy*. This system has no closed-form solution and is solved numerically in Mathcad 10.0 (PTC, Boston, MA, USA). Because the geometry is not known a priori, the system is solved for a set of candidate shape classes; each candidate yields a solution branch, so the procedure returns the admissible bounds of the absorption coefficient $\alpha \left(\lambda \right)$ rather than a single value. These bounds, evaluated at the three wavelengths, locate the particle within the multi-wavelength absorption space introduced in Section 2.4 and therefore within—or outside—the region characteristic of a given polymer.

**Figure 5:**
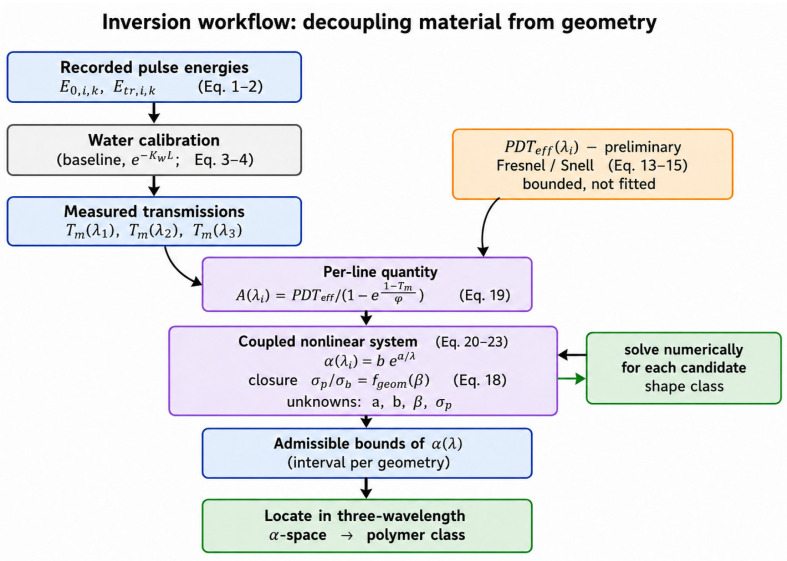
Block diagram of the inversion workflow that decouples material from geometry; the steps and equations are detailed in Section 2.7.

The pulsed multi-wavelength architecture also provides information beyond pulse-energy integration. By matching the repetition frequencies of the three lines, individual and combined two- and three-wavelength pulse forms can be isolated, and the relative time delay and amplitude change in these complex pulse forms carry information that helps to evaluate the real index n and the absorption coefficient α jointly, tightening the bounds returned by the inversion. The complete inversion workflow is summarized in Figure 5.

### 2.8. Experimental Architecture and Diode-Laser Configuration

The experimental architecture implements the measurement regime defined in Section 2.1, Section 2.2, Section 2.3, Section 2.4, Section 2.5, Section 2.6 and Section 2.7. Its purpose is to illuminate the water channel with three selected wavelengths through a common beam geometry, to synchronize the pulse sequence, to measure incident and transmitted pulse energies, and to generate a time-indexed event signature for each particle crossing. The proof-of-concept configuration consists of synchronized pulsed laser-diode modules, pre-sample beam-combining optics, beam-shaping optics, a transparent water channel containing the active sensor volume, three fast photodetectors, a high-speed digital oscilloscope, and a computer for data recording and preliminary processing [42]. Preliminary tests with a parallel reference-beam scheme showed poor reproducibility, traceable to the difficulty of holding the two cuvette windows perpendicular to the beam to better than about one percent; a single-measuring-channel scheme was therefore adopted as the working configuration.

**Figure 6:**
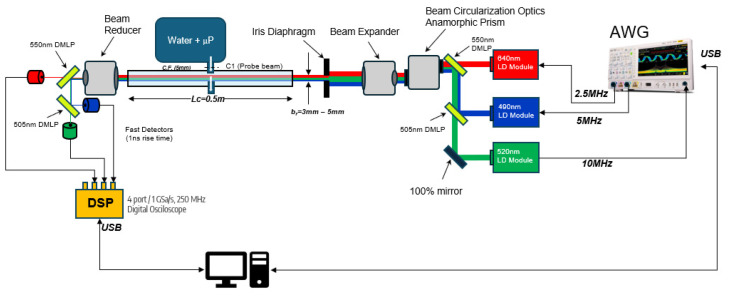
Schematic of the single-measuring-channel proof-of-concept setup; the components and timing are described in Section 2.8.

The main components include an arbitrary waveform generator (SDG1062X, Siglent Technologies, Shenzhen, China) for synchronization and pulse-sequence control; laser-diode modules at 490 nm, 520 nm, and 640 nm (NPL49B, NPL52B, NPL64B; Thorlabs, Newton, NJ, USA); pre-sample beam-combining optics composed of long-pass dichroic mirrors (505 and 550 nm) (DMLP505 and DMLP550; Thorlabs, Newton, NJ, USA) and steering mirrors (Thorlabs, Newton, NJ, USA); beam-shaping optics including anamorphic-prism circularization optics(PS883-A; Thorlabs, Newton, NJ, USA), a beam expander, an iris diaphragm, and a beam reducer; a transparent glass (custom-made) tube used as the water channel and active sensor-volume region; three fast photodetectors (DET10A2, Thorlabs, Newton, NJ, USA; 1 ns rise time), one for each wavelength (490, 520, and 640 nm), for the transmitted pulse signals; a digital oscilloscope for high-speed pulse acquisition (Handyscope HS6-1000, TiePie Engineering, Sneek, The Netherlands); and a PC for data recording and preliminary signal processing. The long-pass dichroic mirrors (505 and 550 nm) combine the three lines before the cell and, as shown in Figure 6, separate the transmitted beam back into its three wavelengths after it, each line reaching its own detector.

Before particle measurements, the reference transmission baseline is established on particle-free water for each wavelength channel, as required by Equation (3). During particle-event acquisition, the three detectors simultaneously record the transmitted pulse on the three wavelength channels, so that the per-wavelength transmissions are obtained from one and the same composite pulse and normalized to this baseline. The selected wavelengths are 490 nm, 520 nm, and 640 nm and the beam diameter is approximately 3 to 5 mm. The arbitrary waveform generator synchronizes the three diode modules in time and overlays their pulses precisely into composite pulse structures: a single-wavelength pulse, a two-wavelength pulse, and a three-wavelength pulse, in which one, two, or all three wavelengths are placed into one and the same pulse envelope. In the three-wavelength composite, the three lines therefore illuminate the particle simultaneously and along the same path, so that all three wavelengths interact with the same particle in the same position and orientation during a single pulse; the pulse duration is approximately 40 ns, short enough to limit particle reorientation during this interaction while allowing reliable pulse-energy integration. The composite structures recur at different rates set by the generator—the three-wavelength composite at the lowest rate (2.5 MHz), the two-wavelength composite at 5 MHz, and the single-wavelength reference at 10 MHz—so that the different structures are distinguished by their repetition rate rather than by post-sample optical filtering. The quantitative measurement used for the inversion is taken from the three-wavelength composite pulse at this lowest repetition rate: because its three lines act on the particle at the same instant, the normalized transmissions $T_{m}\left(\lambda_{1}\right)$, $T_{m}\left(\lambda_{2}\right)$, and $T_{m}\left(\lambda_{3}\right)$ correspond to one and the same projected cross-section $\sigma_{p}$ and optical length $d_{op}$, which is precisely the condition that allows the material contribution to be separated from the geometry in Section 2.7. The single- and two-wavelength composites provide references for baseline and consistency checks. These parameters should be interpreted as a proposed proof-of-concept configuration rather than as fixed optimized values; pulse duration, optical power, repetition frequency, and detector bandwidth should be optimized in a future prototype according to the required flow-velocity range, sensor-volume geometry, and signal-to-noise ratio.

The number of discrete three-wavelength measurements acquired during one crossing event is

(24) $$N_{meas}=t_{cross}\cdot f_{rep}$$

where $t_{cross}$ is the particle crossing time through the active sensor volume and $f_{rep}$ is the repetition frequency of the combined three-wavelength structure (about 2.5 MHz in the present configuration). The crossing time depends on beam diameter, sensor-volume geometry, particle trajectory, and water-flow velocity, and a higher repetition frequency increases the number of pulse-indexed samples available to capture orientation-dependent changes in projected area and optical length. For realistic crossing times and the combined three-wavelength repetition rate, this yields from several hundred to several thousand discrete three-wavelength measurements per crossing event, far more than required, so that only a representative subset need be retained for fast statistical processing. The complete event response is therefore a time-indexed multispectral sequence of the normalized transmissions $T_{m}\left(\lambda_{i},k\right)$ and pulse energies $E_{tr,i,k}$ acquired during the crossing; pulse-shape descriptors such as amplitude, width, and relative timing may be extracted from the same recorded signals to supplement this sequence [36,42].

### 2.9. Microscope Validation and Classification Strategy

The inversion of Section 2.7 is validated against independent geometrical measurements. Reference particles of the most common polymers—LDPE, HDPE, and PP—with sizes between approximately 0.5 mm and 5 mm are characterized in advance by optical microscopy, which provides the projected cross-section and an estimate of the optical length along the beam direction. The same particles are then measured optically, the nonlinear system is solved, and the recovered absorption bounds are compared with the microscope-based geometry to assess how closely the model reproduces the known particle. Repeating this over many particles of each polymer populates the per-polymer regions in multi-wavelength absorption space described in Section 2.4 and provides the calibration against which unknown particles are later evaluated. The present model-based analysis is developed for the three principal polyolefins (LDPE, HDPE, PP), which share smooth polyolefin Urbach behavior and are the dominant environmental contaminants, making them the natural first test of the discrimination axis. Poly(ethylene terephthalate) (PET), an aromatic polyester, has a qualitatively different absorption at the blue edge of the visible range that departs from the smooth polyolefin Urbach tail assumed here; it is therefore not an additional curve under the same parameters but a distinct regime requiring its own parameterization. PET has nonetheless been characterized experimentally alongside the polyolefins, with cross-sections and lengths determined independently by optical microscopy, and is clearly distinguishable from them [29]; its full model-based treatment, together with an appropriate absorption-edge parameterization for aromatic polymers, is the subject of the experimental follow-up. Broadening the material set in this way is part of the planned calibration-database work.

The deployment-stage classification is calibration-driven. Full real-time inversion of every parameter for every pulse is not required in the field; instead, an unknown particle event is assigned to the polymer region it falls into, using the bounds and reference regions established offline. In this sense, the term real-time refers to real-time-capable event assignment based on pre-established calibration regions, while the numerical inversion that builds those regions is performed during calibration and analysis rather than continuously on the device.

## 3. Model-Based Results

The results reported here are analytical and model-based: the framework of Section 2 is exercised with representative, experimentally grounded optical parameters for three common polyolefins to demonstrate that the inversion is well posed and that the polymers separate in the recovered absorption space. These representative absorption coefficients follow the experimental absorption characterization of the same polymers carried out with the group’s multiwavelength laser source on individual particles in filtered water [29], and their order of magnitude is consistent with the complex refractive index disclosed in the patented method [43] through α = 4πκ/λ; the diode wavelengths adopted here (490, 520, and 640 nm) probe the same Urbach-type dispersion at nearby spectral points. The coefficients recovered here represent the intrinsic, single-particle material absorption along the optical path, baseline-corrected against particle-free water, which is distinct from the mass-specific, scattering-affected suspension absorption reported for microplastic ensembles [21]; the two quantities are therefore not directly comparable in magnitude. Experimental verification on real particles is the subject of separate work; the purpose here is to establish that the concept is internally consistent and discriminating before instrument-level validation.

Applying the forward model of Equations (6)–(15) with visible-range refractive indices for low-density polyethylene (LDPE), high-density polyethylene (HDPE), and polypropylene (PP) [38] gives the values summarized in Table 1. The probability of direct transmission lies around 0.85–0.90 and varies only weakly between the polymers, consistent with a realistically rough particle surface, confirming the expectation of Section 2.5 that $PDT_{eff}$ can be supplied to the inversion as a bounded preliminary quantity rather than as a free parameter. The absorption coefficients, by contrast, vary far more strongly: PP is set well below the two polyethylenes at all three lines, whereas LDPE and HDPE are close at the shorter wavelengths and diverge toward the red.

**Figure 7:**
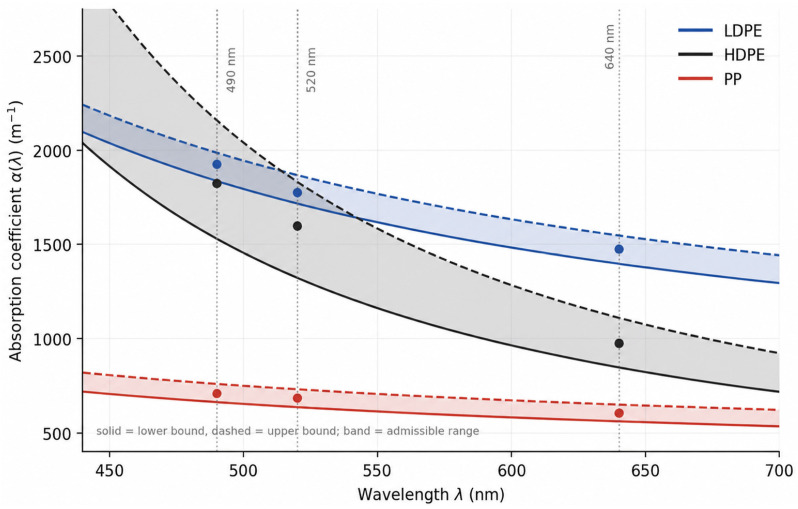
Absorption dispersion for LDPE, HDPE, and PP. For each polymer, the solid and dashed curves are the lower and upper bounds of the admissible range, and the shaded area is the band; filled markers are the band midpoints at the three measurement wavelengths (Table 1). The LDPE and HDPE bands overlap near 490 and 520 nm and separate toward 640 nm, while PP stays well below.

The corresponding absorption dispersion is shown in Figure 7. Each polymer occupies a bounded band rather than a single curve, reflecting the spread across data sources, ageing, and surface state. PP is well separated from the two polyethylenes at all three wavelengths by its markedly lower absorption. LDPE and HDPE—although chemically identical and therefore difficult to separate by their primary molecular vibrational fingerprint—overlap substantially near 490 and 520 nm but separate progressively toward 640 nm, where the LDPE band lies well above the HDPE band. The discrimination is therefore wavelength-dependent: the longer-wavelength channel carries most of the LDPE/HDPE separation, and it is the combination of all three lines, taken as a bounded region in the three-wavelength absorption space, that distinguishes the two polyethylenes that standard vibration-based workflows do not routinely resolve.

**Table 1:** Direct-transmission probability and Urbach-tail absorption coefficient at the three measurement wavelengths for representative polyolefins. Values are representative, model-derived figures consistent with the dispersion curves of Figure 7; α is reported as the midpoint of the admissible band, with the band range given in parentheses. These α values follow the experimental absorption characterization of the same polymers reported in [29], and their order of magnitude is consistent with the complex refractive index disclosed in [43] via α = 4πκ/λ. Refractive indices are literature values for the visible range [38] (PP, 1.49; LDPE, 1.51; HDPE, 1.54).

Polymer	$n_{p}$	$PDT_{eff}$	$\alpha \left(490\right)$ [$m^{-1}$]	$\alpha \left(520\right)$ [$m^{-1}$]	$\alpha \left(640\right)$ [$m^{-1}$]
LDPE	1.51	0.895	1920 (1850–2000)	1780 (1700–1850)	1480 (1400–1550)
HDPE	1.54	0.893	1820 (1550–2100)	1600 (1300–1900)	980 (850–1100)
PP	1.49	0.856	710 (660–760)	680 (640–730)	600 (560–650)

**Figure 8:**
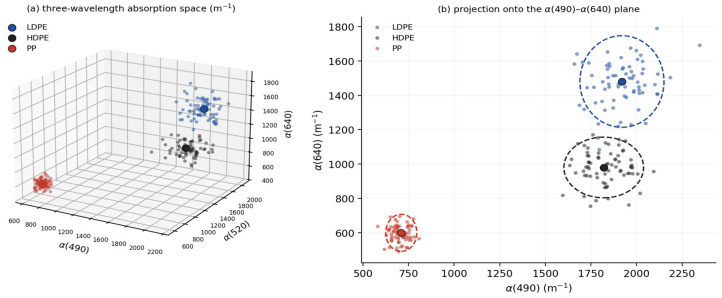
Per-polymer regions in the three-wavelength absorption space, from the modeled values of Table 1: (**a**) three-dimensional view in the α490, α520, and α640 axes and (**b**) projection onto the α490–α640 plane; large markers are the central values.

When the procedure is reversed—generating synthetic water-calibrated transmissions $T_{m}\left(\lambda_{i}\right)$ from these parameters for an assumed particle geometry and then solving the nonlinear system of Equations (19)–(23)—the inversion returns the absorption parameters $\left(a,b\right)$, and hence $\alpha \left(\lambda \right)$, within bounds set by the candidate shape class. Because the geometry is solved jointly through the closure of Equation (18), the recovered $\alpha \left(\lambda \right)$ is not a single value but an admissible interval whose width reflects the uncertainty in shape and orientation. Locating that interval in the absorption space of Figure 8 assigns the particle to the polymer region it occupies, which is the operational basis for classification. The per-polymer regions in this three-wavelength absorption space, populated from the modeled values, are shown in Figure 8. PP is clearly separated by its lower absorption, while LDPE and HDPE occupy distinct, partly adjacent regions despite being chemically identical—the proof-of-concept modeled separation that the method targets. The spread assigned to each region represents micro-disorder, impurities, geometry, and ageing.

### Sensitivity and Uncertainty of the Inversion

The results of the previous section establish that the inversion is internally consistent and that the three polymers separate in the noise-free absorption space. To assess whether this separation survives realistic measurement conditions, the coupled forward-and-inverse model was exercised in a Monte-Carlo framework in which the synthetic transmissions were perturbed before inversion, so that the data are no longer analyzed under the exact conditions that generated them.

**Figure 9:**
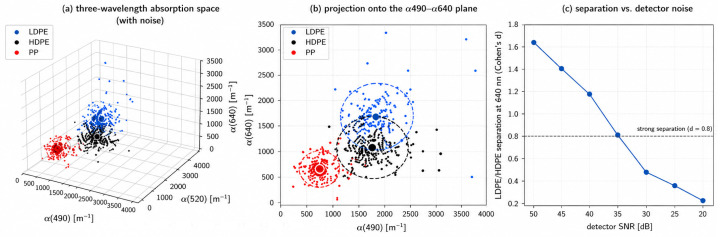
Monte-Carlo uncertainty analysis of the recovered absorption, with detector noise, laser-power fluctuation, PDTeff mis-specification, and particle-to-particle variability propagated through the inversion. (**a**) Three-dimensional view in the α(490), α(520), and α(640) axes; (**b**) projection onto the α(490)–α(640) plane, with dashed 2σ guides; large markers are the central values. (**c**) LDPE/HDPE separation at 640 nm (Cohen’s d) versus detector signal-to-noise ratio, showing that the discrimination remains strong for realistic noise and degrades gracefully below about 35 dB.

Four error sources were propagated: additive detector noise at a representative signal-to-noise ratio; pulse-to-pulse laser-power fluctuation of the order of one percent; mis-specification of the pre-computed PDTeff within its plausible band; and particle-to-particle variability, with the absorption parameters drawn from within each polymer’s admissible band. The particle geometry (projected cross-section and optical length) was taken as known, consistent with the calibration regime in which it is determined independently by optical microscopy. For each polymer, an ensemble of noisy events was generated and inverted, and the recovered α(λ) values were plotted as clouds in the three-wavelength absorption space (Figure 9).

The per-polymer clouds remain separated once these uncertainties are included. PP is clearly resolved from the two polyethylenes at all realistic noise levels. The LDPE/HDPE separation is carried predominantly by the 640 nm channel, where the two bands separate strongly once the per-pulse detector uncertainty (below about 3%, corresponding to a single-pulse signal-to-noise ratio near 30 dB) is reduced by the pulse averaging inherent in the measurement (the source operates at a pulse-repetition frequency of the order of 10 kHz and the transmission is integrated over about one second per event), while at 490 nm, they remain essentially indistinguishable, consistent with Table 1. As shown in Figure 9c, the separation depends on the effective signal-to-noise ratio: it remains strong for an effective ratio at or above roughly 35 dB and is progressively lost below about 30 dB. Because the single-pulse ratio (near 30 dB) is raised to an effective value well above 35 dB by averaging even a modest number of pulses, the predicted LDPE/HDPE discrimination exceeds realistic experimental uncertainty under the measurement conditions used, provided the PDTeff band is held within the stated limits. Preliminary absorption measurements on real polymer particles of these polyolefins, whose cross-sections and lengths were determined independently by optical microscopy, are consistent with this behavior and are reported separately [29]; their full experimental treatment is the subject of the companion paper.

Finally, the event-based logic supports the central device idea: a particle crossing the illuminated volume generates a sequence of pulse measurements (Equation (24)), allowing a time-resolved analysis of the changing projected cross-section and optical length rather than a single-shot estimate. This distinguishes the architecture from a static transmission measurement and is particularly relevant in real aquatic environments, where particles are mobile and their projected optical response varies during transit.

## 4. Discussion

The principal contribution of the present work is the formulation of a physically grounded and experimentally realistic concept for compact in situ optical monitoring of microplastics in water, protected by European patent EP4196764 (granted 6 August 2025) [43], together with an explicit inversion scheme that separates material from geometry. The method does not reproduce the full chemical specificity of FTIR, Raman spectroscopy, or Py-GC/MS, and it is positioned as a complementary approach for rapid field screening, continuous observation, and event detection directly in the water path [21,22,23,24]. Laboratory methods remain essential for validated polymer identification, but their preparation requirements, automation constraints, and time demands limit their suitability for real-time aquatic deployment [9,10,11,12,14,15,17]; even routine-oriented automated μ-Raman protocols remain constrained by image-analysis performance, stage precision, blank correction, and total analysis time [17].

At the same time, the method offers a discrimination axis that standard vibration-based workflows do not routinely provide. Because it measures the sub-gap absorption tail rather than molecular vibrations, it is sensitive to the band-gap and structural state of the polymer, and the worked example of Section 3 shows that it can in principle separate structural modifications of identical chemical composition, such as LDPE and HDPE. This is a complementary capability—supported here by the model and to be confirmed by the experimental verification reported separately—rather than a replacement for chemical confirmation.

The concept is also distinct from other optical approaches to in situ microplastic detection. Fluorescence-based quantification relies on staining or intrinsic emission and does not by itself yield the polymer class [24]; transmission- and scattering-based sizing–identification schemes recover the particle size and count but provide limited polymer specificity [27]; and reported portable optical sensors demonstrate detection of a particle presence in freshwater without resolving the polymer type [25,26]. The present method adds a polymer- and structure-discrimination axis on top of detection and sizing while remaining a transmission-only, label-free, and compact architecture [22,23]. Table 2 summarizes this comparison with representative optical sensors and established laboratory methods.

Two elements of the model deserve emphasis because they carry the method. The probability of direct transmission is not an ad hoc parameter: Equations (13)–(15) derive it from the Fresnel relations, with its substance dependence entering through the refractive-index contrast and its geometry dependence entering through the averaging over the surface-angle distribution, so that it can be bounded in advance and supplied to the inversion as a preliminary quantity. The decoupling of size and form, through the effective optical length of Equation (16) and the geometry closure of Equation (18), is what makes the nonlinear system well posed for a given candidate shape and what justifies reporting absorption bounds rather than a single value.

The concept has limitations that must be recognized explicitly. The transmission-only response does not provide chemical confirmation of the polymer identity and should be considered complementary to FTIR, Raman, and Py-GC/MS rather than a replacement. Natural-water matrices may introduce interference from mineral particles, organic matter, air bubbles, turbidity, biofilms, and aged or aggregated microplastics, so the classification performance depends on baseline correction and on the representativeness of the calibration regions with respect to polymer type, size, shape, surface condition, ageing state, and water-matrix properties. Two mechanisms mitigate the calibration-drift concern intrinsic to any baseline-referenced method. First, a discrete particle crossing produces a transient deficit that is structured and correlated across the three lines, which is distinguishable from the slow, spectrally smooth drift caused by turbidity or dissolved organic matter; this difference supports periodic re-referencing of the particle-free baseline during operation. Second, strongly scattering air bubbles and aggregates drive the extinction efficiency toward unity across all three wavelengths and are therefore flagged as non-invertible events and rejected rather than misclassified as polymers. Furthermore, ageing, oxidation, and additives act on the band-gap, the Urbach parameters, and the surface state, so they broaden each polymer’s region in absorption space rather than shifting it arbitrarily; the calibration database must consequently be populated with aged, weathered, and additive-bearing reference particles representative of the target waters. The sphere, disk, and cylinder classes used here are candidate closure relations adopted to keep the inversion well posed, not an assertion that real particles take these shapes; they are a first approximation that does not span the full morphological diversity of environmental particles. Irregular fragments, fibers, and aggregates fall outside this basis and will require an extended shape library or data-driven shape inference, which is the aim of a planned time-domain (LTI/convolution) formulation designed to handle arbitrary morphology. The inversion returns bounds rather than exact values, and the width of these bounds depends on the quality of the $PDT_{eff}$ estimate, on the assumed shape classes, and on the achievable signal-to-noise ratio. Future work should focus on expanded calibration datasets, validation in real environmental matrices, systematic baseline correction, and direct comparison with established reference techniques, with particular attention to aged particles, variable surface roughness, biofilm formation, and mixed suspended-particle backgrounds [10,11,12,14,15,17,19,21,22,23,24,28,44].

## 5. Conclusions

This work establishes the theoretical and methodological basis of a compact optical approach for real-time, in situ assessment of suspended microplastics in water, and it is a concept-and-theory study whose experimental validation is the subject of separate work. The measurement principle is already embodied in a laboratory realization at technology-readiness level TRL-4 built from commercially available components, and the accompanying experimental results are reported in a separate companion paper. The core result is that, after calibration on particle-free water, a single particle event reduces to a water-normalized transmission whose deficit separates into a geometrical beam–particle overlap and a wavelength-dependent extinction efficiency, and that this coupling can be inverted: solving a coupled nonlinear system over three synchronized visible wavelengths recovers the bounds of the polymer absorption coefficient while decoupling particle size and shape. Because each polymer occupies a bounded region in multi-wavelength absorption space fixed by its band gap and structural state, the method offers, in principle, a discrimination capability that extends to structural modifications of identical chemical composition, such as LDPE and HDPE—a separation that is difficult for standard vibration-based workflows. Built around three synchronized visible laser-diode lines as a deliberate compromise between information content and device simplicity, the approach is positioned not as a competitor to laboratory spectroscopy but as a complementary field-oriented screening and monitoring layer. Future work should focus on expanded calibration, validation in real waters, direct comparison with established reference methods, and progressive miniaturization toward a deployable prototype.

## Figures and Tables

**Table 2:** Comparison of the proposed multispectral pulsed-transmission concept with representative optical microplastic sensors and with established laboratory reference methods. Size ranges are indicative and depend on configuration; “limited LDPE/HDPE” denotes that standard vibration-based workflows classify both as polyethylene and resolve the structural difference only with additional crystallinity-sensitive analysis. The accuracy of the present concept is pending the experimental validation reported in the companion paper.

Method/Source	Principle	Size Range	Measurand	Polymer Discrimination	In Situ/Real Time
This work (concept)	Multispectral pulsed transmission, three diode lines (490/520/640 nm) + model inversion	~250–300 µm to 5 mm (extendable to ~100 µm)	Polymer + structural class, size, form	Yes—incl. LDPE/HDPE	Yes/yes, label-free (accuracy pending validation)
Asamoah et al. [25]	Specular reflection + transmission, single 635 nm laser	MP range (~1–5 mm)	Presence of transparent/translucent MPs	No	Portable/yes, label-free
Campos-López et al. [26]	Portable multi-wavelength optical sensor	MP range	Detection + calibration	Limited	Portable/yes, label-free
Glöckler et al. [27]	Elastic light scattering + Raman in flow cuvette	~4–100 µm (PS/PMMA)	Size + material, single step	Yes (via Raman)	Flow-through/proof of principle
Nicolai et al. [24]	Real-time fluorescence particle counter	MP range (counter)	Quantitative count	No (density-matched, not polymer ID)	Real-time/needs staining or emission
FTIR/µFTIR [9,10,11,12]	Vibrational IR absorption spectroscopy	~10–20 µm to 5 mm	Polymer chemical ID, morphology	Yes (chemical); limited LDPE/HDPE	No—laboratory, sample preparation
Raman/µRaman [9,10,11,12,13,17]	Vibrational Raman scattering	~1 µm to 5 mm	Polymer chemical ID, morphology	Yes (chemical); limited LDPE/HDPE	No—laboratory, slow, fluorescence-prone
Py-GC/MS [9,13]	Thermal decomposition + mass spectrometry	Bulk/mass-based	Polymer mass, additive ID	Yes (chemical); destructive	No—laboratory, destructive

## Data Availability

The original contributions presented in this study are included in the article. Further inquiries can be directed to the corresponding authors.

## Abbreviations and Nomenclature

LDPE	Low-density polyethylene
HDPE	High-density polyethylene
PP	Polypropylene
$\alpha \left(E\right),\;$ $\alpha \left(\lambda \right)$	Absorption coefficient as a function of photon energy and of wavelength
A, ΔE, $E_{U},\;$ E	pre-exponential coefficient, effective absorption-edge energy, Urbach energy, and photon energy in the Urbach-tail expression
a, b	Wavelength-form Urbach fitting parameters (spectral slope and magnitude of $\alpha \left(\lambda \right)$)
h, c, λ	Planck constant, speed of light in vacuum, and optical wavelength
n, $n_{p},\;$ $n_{w}$	Real part of the refractive index, polymer index, and water index
κ	Imaginary part of the refractive index (extinction)
$P_{0},\;$ $P_{tr},\;$ $E_{0},\;$ $E_{tr},\;$ $t_{p}$	Incident and transmitted pulse power, incident and transmitted pulse energy, and pulse duration
$K_{a}\left(\lambda \right),\;$ L	Water attenuation coefficient and optical path length through the water channel
$T_{w},\;$ $T_{m}$	Water-only transmission and water-calibrated normalized (measured) transmission
$\sigma_{p},\;$ $\sigma_{b},\;$ $r_{b},\;$ $r_{p}$	Projected particle cross-section, beam cross-section, beam radius, and particle radius
x	Size parameter, x=πd/λ
$Q_{ext}$	Extinction efficiency of the particle ($f_{Q}$ of n, $\alpha \left(\lambda \right),\;$$d_{op}$)
$PDT_{eff},\;$ $R_{en},\;$ $R_{ex},\;$ $\theta_{n},\;$ $\theta_{x},\;$ $\theta_{en},\;$ $\theta_{ex}$	Direct-transmission probability, entry/exit Fresnel reflectances, and incidence/refraction angles
$d_{op},\;$ $V_{p},\;$ d, p, asy, β	Effective optical length, particle volume, transverse and longitudinal dimensions, asymmetry parameter, and the effective optical length entering the inversion (β ≡ d_op)
φ	Effective beam–particle overlap (filling) factor in the inversion
$A\left(\lambda_{i}\right)$	Per-line intermediate quantity used in the inversion
$N_{meas},\;$ $t_{cross},\;$ $f_{rep}$	Number of measurements per event, crossing time, and repetition frequency
